# Performance Comparison of Proton Exchange Membrane Water Electrolysis Cell Using Channel and PTL Flow Fields through Three-Dimensional Two-Phase Flow Simulation

**DOI:** 10.3390/membranes12121260

**Published:** 2022-12-13

**Authors:** Seongsoon Park, Woojung Lee, Youngseung Na

**Affiliations:** Department of Mechanical and Information Engineering, University of Seoul, Seoul 02054, Republic of Korea

**Keywords:** PEMWE, flow field, two-phase flow simulation, bubble overvoltage, channel geometry, PTL properties, gravity, flow rate

## Abstract

Water electrolysis technology is required to overcome the intermittency of renewable energy sources. Among various water electrolysis methods, the proton exchange membrane water electrolysis (PEMWE) cell has the advantages of a fast response and high current density. However, high capital costs have hindered the commercialization of PEMWE; therefore, it is important to lower the price of bipolar plates, which make PEMWE expensive. In addition, since the flow field inscribed in the bipolar plate significantly influences the performance, it is necessary to design the enhanced pattern. A three-dimensional two-phase flow model was used to analyze the two-phase flow and electrochemical reactions of the PEMWE anode. In order to compare the experimental results with the simulation, experiments were conducted according to the flow rate, and the results were in good agreement. First, as a result of comparing the performance of the channel and PTL (porous transport layer) flow fields, the channel flow field showed better performance than the PTL flow field. For the channel flow field, the higher the ratio of the channel width-to-rib width and the permeability of PTL, the performance got better. In the case of the PTL flow field, with the increased capillary pressure, the performance improved even if the PTL permeability decreased. Next, the direction of gravity affected the performance only when the channel flow field was used, and the X+ and Z+ directions were optimal for the performance. Finally, increasing the inlet flow rate could reduce the difference in performance between the channel and PTL flow fields, but the pressure drop gradually increased.

## 1. Introduction

Over the past few decades, increased carbon emissions have been warming the Earth at an unprecedented rate. Global carbon dioxide emissions were expected to triple from 1975 to 2020, highlighting the urgent need for a zero-carbon energy infrastructure [1]. Therefore, the contribution of renewable energy sources to reduce carbon emissions, such as wind power and solar power, are increasing every year in the global energy supply. Despite significant research and development on renewable energy technologies, such as wind and solar energy, integrating these energies into the electrical grid remains challenging due to the intermittent availability of these energy sources [2]. Therefore, the storage of renewable energy is necessary to overcome the intermittent supply [3].

Hydrogen is one of the most promising solutions to this intermittent problem, as an energy carrier, because it can be converted from electricity to hydrogen. When it is converted to electricity again, it emits only water as a byproduct, without carbon emissions [4]. The advantages of hydrogen over fossil fuels include its non-toxicity, high mass-energy density, and high energy efficiency (>70%) [5]. Currently, 96% of the world’s produced hydrogen-using fossil fuels are called grey hydrogen [6]. The fossil-fuel-based hydrogen production method is a transitional technology used to achieve carbon-free hydrogen production. Therefore, the ultimate hydrogen production method in the future is water electrolysis, called green hydrogen, with the lowest carbon emissions.

Water electrolysis systems are typically divided into three types: alkaline water electrolysis cells (AEC), proton exchange membrane water electrolysis cells, and solid oxide water electrolysis cells (SOEC). From a technical point of view, AEC are now well developed enough to produce renewable hydrogen at a significant rate, whereas SOEC, which produce hydrogen from steam, are still in the laboratory stage. Compared with other water electrolysis methods, the biggest advantages of PEMWE are its fast system response, in the millisecond level, and working at high current densities above 2 A/cm^2^. These advantages are even more pronounced when related to renewable energy sources, which are characterized by an intermittent power supply [7].

However, the high capital cost hinders the commercialization of PEMWE. A total cost analysis of the 1 MW PEMWE showed that stack components accounted for 45% of the total cost, of which 53% was the bipolar plate and 17% was the PTL [8]. Bipolar plates are typically composed of titanium and can withstand acidic environments, which increases their cost [9]. In addition, the bipolar plate has a flow field carved into the plate surface, which can distribute the reactants uniformly within the active area and effectively remove the products from the active area via the PTL. The flow field design of bipolar plates has a significant impact on water electrolysis performance [10]. A well-designed flow field ensures uniformity of fluid flow and pressure distribution within the PTL, enabling operation at high pressures and current densities [11]. When operated at a high current density, the mass transport loss becomes dominant and efficiency reduces [12,13,14]. To reduce these mass transfer losses, an appropriate mass transfer strategy is essential to supply enough reactants and remove products [15]. In addition, the heat generated at high current densities remains trapped in the stack, which can degrade the performance and lifespan of the PEMWE [16]. Therefore, it is necessary to study the flow field design to reduce the cost of the anode plate, even at high current density operation, and to systematize the current density distribution without mass transport loss.

To optimize the flow field design at high current densities, many scholars have numerically and experimentally investigated the effect of the high current density behavior and design of the flow field on the PEMWE. Immerz et al. measured the local distribution of current density and electrochemical impedance spectroscopy (EIS) along a 50 cm PEMWE with a high and low stoichiometric water ratio [17]. The results show that for the stoichiometric water ratio of less than five, the mass transport and ohmic voltage losses increase significantly. Olesen et al. developed a three-dimensional model to investigate three different circulars: interdigitated anode flow fields operated at high pressure and high current density. In all three cases, the occurrence of hotspots was associated with a poor distribution of two-phase flow and current density [18]. They also found that the same land width between channels provided the best charge, mass, and heat distribution. Nie et al. numerically simulated and experimentally measured three-dimensional pressure and velocity distributions of a parallel flow field [19]. They found that the pressure decreased from the inlet to the outlet and that the velocity and temperature distributions were not uniform along the channel. Majasan et al. correlated the electrochemical performance to the two-phase flow visualization of parallel and serpentine flow fields [20]. The results showed that the annular flow regime degrades cell performance. Ruiz et al. conducted a computational study of parallel, serpentine, and multi-serpentine channel geometries to compare the performance of the PEMWE [21]. They found that the serpentine channel design exhibited the highest performance despite the high pressure drop in the channel.

However, the effect of the channel geometry of the serpentine flow field on the performance of the PEMWE is yet to be explored. On the other hand, if the flow field is not engraved on the bipolar plate, the price of the bipolar plate can be drastically lowered; therefore, research on the performance of PTL flow field cells is required. In addition, studying the effect of operating conditions, such as the PTL properties, gravity direction, and flow rate, on the performance is necessary. Changing the PTL characteristics, such as structure, composition, thickness, and wettability, is expected to enhance the performance of the PEMWE [22]. Therefore, this study compares the performance of the channel and PTL flow fields and investigates the effects of channel geometry, PTL properties, gravity, and flow rate on the performance of the PEMWE using a three-dimensional two-phase flow simulation and experimental validation. The results of this study will help determine the possibility of PEMWE with a PTL flow field and suggest ways to improve the performance of the PEMWE by adjusting the channel geometry, PTL properties, gravity, and flow rate. Moreover, the findings of this study help understand the current density distribution and two-phase flow inside the cell.

## 2. Methodology

### 2.1. Simulation Domain

**Figure 1:**
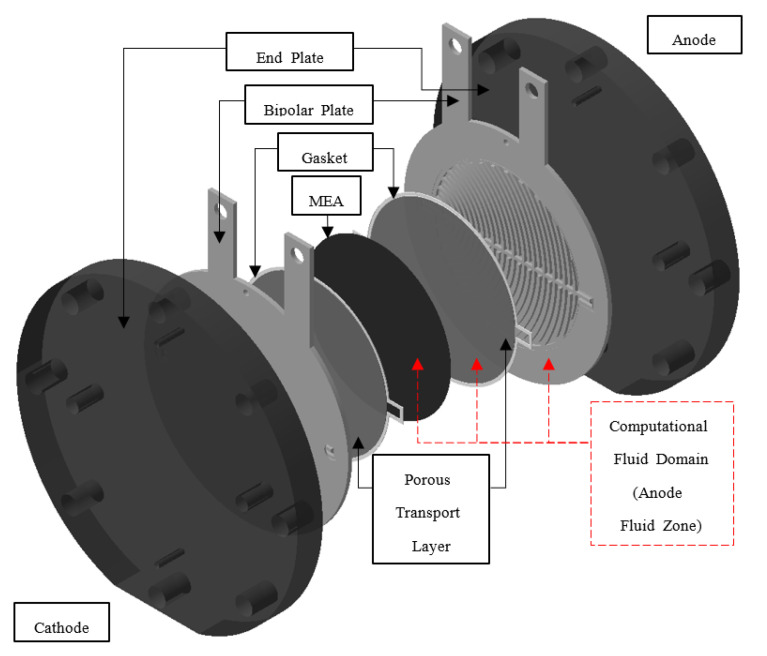
Schematic of the PEMWE and computational fluid domain used for this study.

In this study, the circular PEMWE was investigated, and the diameter of the circular active area was 11.284 cm with an area of 100 cm^2^. Figure 1 shows a schematic of the PEMWE and computational fluid domain used in this study. This single cell consisted of an anode and cathode end plates and two titanium bipolar plates coated with platinum, PTLs, gaskets, and membrane electrode assemblies (MEA), each. The MEA consisted of Nafion 117 and Ir- and Pt-based catalysts for the anode and cathode sides, respectively (Fumatech, Bietigheim-Bissingen, Germany). Titanium paper was used as the anode PTL (2GDL06N-025, BEKAERT, Zwevegem, Belgium). The PTL on the cathode side was composed of carbon paper (TGP-H-120, Toray, Tokyo, Japan). The gaskets on both sides were composed of silicone (CNL Energy, Seoul, Republic of Korea). To study the effect of the two-phase flow of water and oxygen generated in the anode flow field on the performance of the PEMWE, the anode channel, PTL, and MEA were set as the simulation domains. The anode channel and PTL were composed of grids for calculating the electrochemical reactions and two-phase flow, and the MEA was composed of a mathematical function for calculating the ohmic resistance, not grids. In the PEMWE, water oxidizes at the anode of the PEMWE to produce oxygen, and protons are reduced at the cathode to produce hydrogen. The half-cell reactions used are as follows [23]:

(1) $$H_{2}O\to \;\frac{1}{2}O_{2}+2H^{+}+2e^{-}\;\left(\mathrm{Anode}\right)$$

(2) $$2H^{+}+2e^{-}\to H_{2}\;\left(\mathrm{Cathode}\right)$$

**Figure 2:**
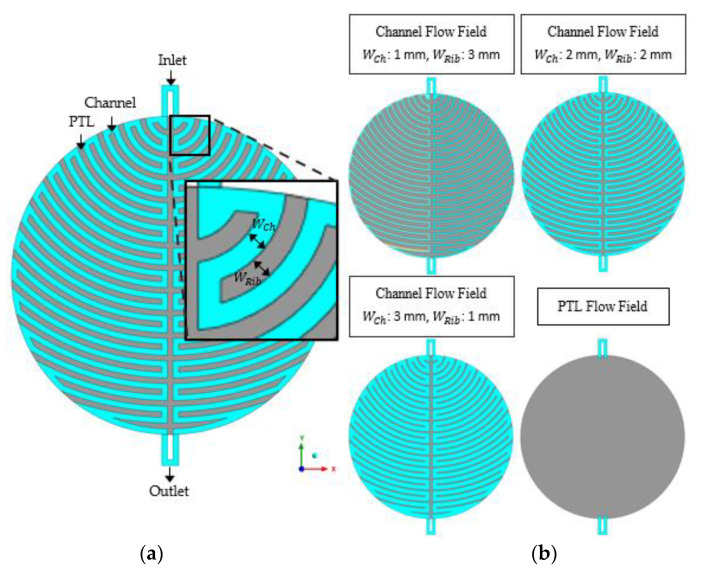
Schematic of (**a**) the computational fluid domain and (**b**) channel and PTL flow fields in the anode side of PEMWE.

**Figure 3:**
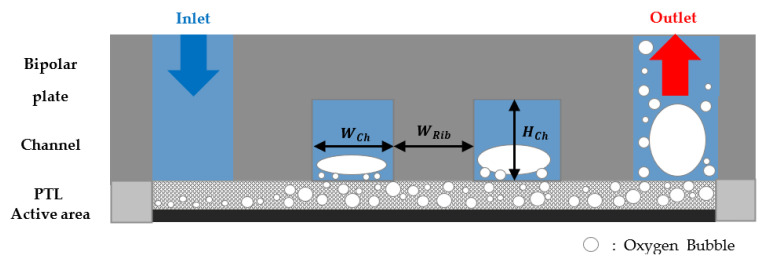
Schematic of the anode channel geometry with the channel flow field in the bipolar plate for PEMWE.

Figure 2 shows a schematic of the computational fluid domain and channel and PTL flow fields, and Figure 3 shows a schematic of the anode channel geometry with the channel flow field in a bipolar plate. In the case of the channel flow field, the anode channel geometry consists of *W_Ch_*, *W_Rib_*, and *H_Ch_* in Table 1. The PTL flow field was the same as in the case of *H_Ch_* = 0 mm. The geometric parameters used in the simulations are listed in Table 1.

### 2.2. Model Assumptions

The following assumptions were applied to simplify the model and calculation:

1. The activation voltage of the cathode was ignored because of the fast reaction rate of the cathode [24].
2. The flow through the channel and PTLs were assumed as laminar flow because of the low Reynolds number.
3. It is assumed that the properties of the PTL are isotropic and identical in all porous media regions.
4. The anode was not pressurized and the oxygen flow from anode to cathode was ignored [25].

### 2.3. Governing Equations

To obtain the current density generated by the electrochemical reaction at a given operating voltage, a current density that satisfies the following formula was obtained [26], and the parameters used in the electrochemical model are presented in Table 2:

(3) $$V_{op}=V_{rev}+\eta_{act}+\eta_{ohm}+\eta_{bubble},$$

where $V_{op}$ is the operating voltage, $V_{rev}$ is the reversible voltage, $\eta_{act}$ is the activation overvoltage, $\eta_{ohm}$ is the ohmic overvoltage, and $\eta_{bubble}$ is the bubble overvoltage. The reversible voltage was obtained as a function of temperature using the following equation [27]:

(4) $$V_{rev}=E^{0}+\frac{\Delta s}{nF}\left(T-T_{0}\right),$$

where $E^{0}$ is the reversible voltage in the standard state, n is the number of electrons participating in the reaction, F is Faraday’s constant, Δs is the change in entropy, T is the temperature, and $T_{0}$ is the temperature in the standard state. The activation overvoltage was assumed using the Tafel equation, which is as follows [27]:

(5) $$\eta_{act}=\frac{RT}{\alpha nF}\ln\left(\frac{i}{i_{0}}\right),$$

where α is the transfer coefficient, R is the universal gas constant, i is the current density, and $i_{0}$ is the exchange current density. Here, *an* and $i_{0}$ were obtained by Tafel fitting using the I–V curve of the low current density region. The ohmic overvoltage was obtained by ignoring the electrical resistance and considering only the membrane resistance as follows [27]:

(6) $$\eta_{ohm}=i\times R_{mem},$$

where $R_{mem}$ is the areal resistance of the membrane, which was obtained by integrating the ion conductivity of the membrane with the thickness of the membrane in the direction of thickness, and the ion conductivity of the Nafion 117 membrane was obtained as a function of the water content and temperature. The following equations were used [27]:

(7) $$R_{mem}=\int_{0}^{t_{mem}}\sigma_{mem}dt,$$

(8) $$\sigma_{mem}=\left(0.00514\lambda -0.00326\right)\exp\left(1268\left(\frac{1}{303}-\frac{1}{T}\right)\right),$$

where $t_{mem}$ is the thickness of the membrane, $\sigma_{mem}$ is the ion conductivity of the membrane (S/cm), and λ is the water content of the membrane. The membrane of PEMWE was assumed to be sufficiently hydrated because of contact with water [28,29]. Finally, the bubble overvoltage generated by reducing the original active area of the catalyst was calculated as a function of saturation using the following equation [30,31]:

(9) $$\eta_{bubble}=\frac{RT}{\alpha nF}\ln\left(\frac{1}{1-\alpha_{g}\;}\right),$$

where $\alpha_{g}$ is the saturation of oxygen, which is the volume fraction of oxygen on the active area, and $1-\alpha_{g}$ is equal to $\alpha_{l}$, the saturation of water, which is the volume fraction of water on the active area.

The Eulerian multiphase model, which calculates the conservation equation for each phase, was used to simulate the two-phase flow of water and oxygen. The parameters used in the conservation equations are listed in Table 3. The continuity equations are as follows [32]:

(10) $$\nabla \cdot \left(\varepsilon \alpha_{l}\rho_{l}\vec{v_{l}}\right)=\varepsilon S_{l},$$

(11) $$\nabla \cdot \left(\varepsilon \alpha_{g}\rho_{g}\vec{v_{g}}\right)=\varepsilon S_{g},$$

where ε is the porosity of the porous media and ε=1 for non-porous media, $\alpha_{l}$ and $\alpha_{g}$ are the volume fractions of water and oxygen, respectively. ρ is the density of each phase, $\vec{v}$ is the velocity of each phase, and S is the source term of each phase. The momentum conservation equations are as follows [32]:

(12) $$\nabla \cdot \left(\varepsilon \alpha_{l}\rho_{l}\vec{v_{l}}\vec{v_{l}}\right)=-\varepsilon \alpha_{l}\nabla \left(P-P_{c}\right)+\nabla \cdot \left(\varepsilon {\overset{=}{\tau }}_{l}\right)+\varepsilon \alpha_{l}\rho_{l}\vec{g}+\varepsilon K_{lg}\left(\vec{v_{l}}-\vec{v_{g}}\right)-\alpha_{l}^{2}\varepsilon^{2}\frac{\mu_{l}\vec{v_{l}}}{K},$$

(13) $$\nabla \cdot \left(\varepsilon \alpha_{g}\rho_{g}\vec{v_{g}}\vec{v_{g}}\right)=-\varepsilon \alpha_{g}\nabla P+\nabla \cdot \left(\varepsilon {\overset{=}{\tau }}_{g}\right)+\varepsilon \alpha_{g}\rho_{g}\vec{g}+\varepsilon K_{gl}\left(\vec{v_{g}}-\vec{v_{l}}\right)-\alpha_{g}^{2}\varepsilon^{2}\frac{\mu_{g}\vec{v_{g}}}{K},$$

where P is the pressure and $P_{c}$ is the capillary pressure. ${\overset{=}{\tau }}_{\;}$ is the stress-strain tensor of each phase and $\vec{g}$ is the acceleration of gravity. $K_{lg}$ which is equal to $K_{gl}$, is the interphase momentum exchange coefficient and the symmetric model was used for drag coefficient. μ is the viscosity, K is the permeability of the porous media and the last term, viscous resistance term of the porous media, is 0 for the non-porous media. The PTL properties used in the momentum-conservation equations are listed in Table 4 [33]. The capillary pressure term was applied to the momentum conservation equation of the hydrophilic phase in the porous media, and the above-stated equation pertains to the case where the porous media is hydrophilic. Capillary pressure was obtained using the Leverett J-function, a function of porosity and permeability, and the equation is as follows [34]:

(14) $$P_{c}=\sigma cos\theta_{p}\sqrt{\frac{\varepsilon }{K}}\left[1.417\left(1-\alpha_{p}\right)-2.12{\left(1-\alpha_{p}\right)}^{2}+1.263{\left(1-\alpha_{p}\right)}^{3}\right]\;\left(\theta_{p}\;\leq 90{}^{\circ}\right),$$

where the subscript p is the wetting phase, σ is the surface tension coefficient, and $\theta_{p}$ is the contact angle of the wetting phase. The energy conservation equation are as follows [32]:

(15) $$\nabla \cdot \left(\varepsilon \alpha_{l}\rho_{l}\vec{v_{l}}\left(h_{l}+\frac{v_{l}^{2}}{2}\right)\right)=\nabla \cdot \left(\varepsilon \alpha_{l}k_{eff,l}\nabla T_{l}+\varepsilon {\overset{=}{\tau }}_{eff,l}\cdot \vec{v_{l}}\right),$$

(16) $$\nabla \cdot \left(\varepsilon \alpha_{g}\rho_{g}\vec{v_{g}}\left(h_{g}+\frac{v_{g}^{2}}{2}\right)\right)=\nabla \cdot \left(\varepsilon \alpha_{g}k_{eff,g}\nabla T_{g}+\varepsilon {\overset{=}{\tau }}_{eff,g}\cdot \vec{v_{g}}\right),$$

where h is the enthalpy of each phase, $k_{eff}$ is the effective thermal conductivity of each phase, and ${\overset{=}{\tau }}_{eff}$ is the effective stress-strain tensor of each phase.

### 2.4. Boundary Conditions

The mass flow inlet of water, pressure outlet, and non-isothermal conditions were applied to the boundary conditions, and the no-slip condition and fixed temperature were applied to the wall to simulate the experiments. The parameters used in the boundary conditions are listed in Table 5. The source term of the continuity equation considered the amount of water consumed by the reaction, electroosmotic drag, and the amount of oxygen produced by the reaction and was only applied to cells in the grid adjacent to the active area. In the sink term for water, the term on the left of the parentheses considers consumption by the reaction, and the term on the right considers crossing the membrane by electroosmotic drag. The formulae used are as follows:

(17) $$S_{l}=\frac{-I\;\times \;M_{l}}{V_{cell}}\left(\frac{1}{2F}+\frac{2.5\lambda }{22F}\right),$$

(18) $$S_{g}=\frac{I\;\times \;M_{g}}{4F\;\times \;V_{cell}},$$

where I is the current (A), M is the molecular weight of each phase, and $V_{cell}$ is the volume of the cell in the grids adjacent to the active area (cm^3^). In addition, heat flux from the reaction was applied to the surface of the active area. The formula used is as follows:

(19) $$\dot{q}=\left(V_{op}-V_{Th,\;\;neu}\right)\times i,$$

where $\dot{q}$, the heat flux (W/cm^2^), applied to the active area, and $V_{Th,\;neu}$ is the thermal neutral voltage. In the model, heat flux boundary condition at the active area’s wall is used to determine the active area’s wall temperature adjacent to a fluid grid as:

(20) $$T_{w}=\frac{\dot{q}}{h_{f}}+T_{f},$$

where $T_{w}$ is the active area’s wall surface temperature, $h_{f}$ is the fluid-side local heat transfer coefficient, and $T_{f}$ is the local fluid temperature.

### 2.5. Experimental Setup

**Figure 4:**
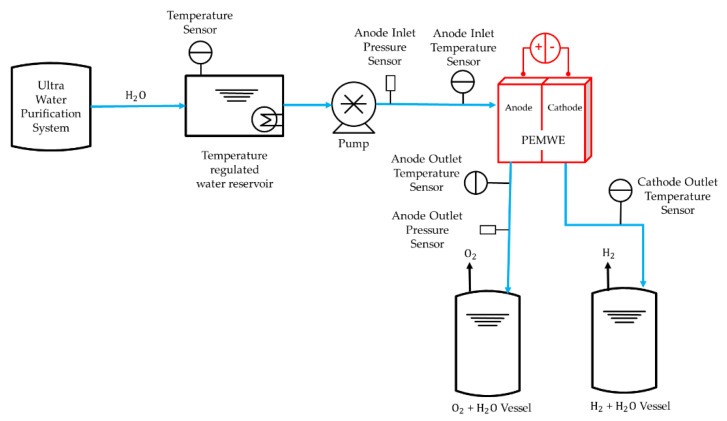
Schematic of the pump, reservoir, and sensors used in the PEMWE experiment procedure for validation.

A PEMWE single cell, with an active area of 100 cm^2^, was fabricated. Endplates, which were composed of aluminum, were 380 cm^2^. The area of titanium bipolar plates was 201 cm^2^, and they were coated with platinum with the thickness was 0.3 μm. The grooves for the gasket and flow field were imprinted on the bipolar palates. The anode flow field pattern used in the models were all identical. The information of membrane electrode assembly, porous transport layers, and gaskets were stated in Section 2.1 simulation domain. A temperature of 25 °C and humidity of 60% were maintained at a laboratory using a thermo-hygrostat (PVU 80T7P1-A, Kiturami, Seoul, Republic of Korea). A PEMWE single cell was assembled using eight M10 bolts by applying a torque of 6.2 N·m. This experiment was conducted in a commercial test station (water electrolysis system, CNL Energy, Seoul, Republic of Korea). It supplied the reactant and exhausted the products. The temperature and pressure were also measured and recorded in the experiment. The tolerances of the temperature sensor (JX-G, Fukuden, Osaka, Japan) was ±2.5 °C and the accuracy of the pressure transmitter (P201GF0003MPCD, Allsensor, Seoul, Republic of Korea) were ≤±0.25% full scale. The specific experiment procedure is shown in Figure 4. Ultra-pure water (18.2 MΩ·cm) was distilled using an ultrapure water purification system (Aquapuri 5 series, Youngin Chromass, Anyang, Republic of Korea). The samples were stored in a reservoir and maintained at a temperature of 80 °C. A constant water flow rate was injected into the anode channel inlet from a diaphragm pump (SIMDOS FEM 1.10, KNF, Freiburg im Breisgau, Germany). The potential of the cell was given by a power supply (DCS12-250E; AMETEK, Berwyn, PA, USA). The hydrogen and oxygen produced by the reaction were exhausted. The temperature of the anode channel inlet was fixed at 55 °C, and the cell temperature was maintained at 55 °C through a heating plate. The polarization curve was measured using chronoamperometry (CA). The voltage was increased in steps of 0.1 V from 1.4 V to 1.8 V. In order to compare the results of the experiment and numerical simulation, experiments were performed at the water flow rates of 5 and 10 sccm.

## 3. Results and Discussion

### 3.1. Model Validation

**Figure 5:**
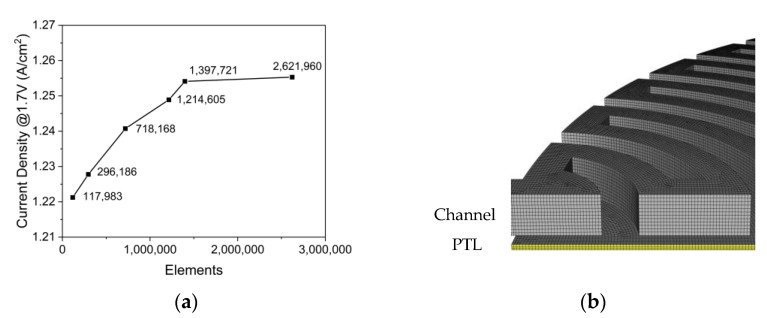
(**a**) Results of the grid independence test of the PEMWE model and (**b**) channel and PTL grids used in the PEMWE simulation (*W_Ch_*: 2 mm, *W_Rib_*: 2 mm, *H_Ch_*: 2 mm, *ε*: 0.75, *K*: 30.2 × 10^−12^ m^2^, *θ*: 30°, ${\dot{Q}}_{in}$: 10 sccm, *T_in_*: 328.15 K, *T_cell_*: 328.15 K, *V_op_*: 1.7 V and $\vec{g}$: z+).

We numerically investigated the simulations using the commercial CFD software ANSYS Fluent 2021R2 (Ansys, Canonsburg, PA, USA) with user-defined functions. The computational resource for this study was Intel (R) Xeon (R) CPU E5-2690 v2 @ 3.00GHZ (40 CPUs) (Intel, Santa Clara, CA, USA) and 131072MB RAM. The two-phase flow was solved using a coupled pressure, velocity method. To capture the gradients of physical values in grids, as the spatial discretization method, PRESTO! was used for pressure, the first order upwind was used for momentum, volume fraction, and temperature. To stabilize the steady-state solver, the simulation was performed by reducing the pseudo-time step from 1 to 0.01. To determine the convergence of the simulation model, the simulation was conducted until the residual of the continuity equation, momentum conservation equation, volume fraction equation was lower than 1.0 × 10^−4^, and the residual of the energy conservation equation was lower than 1.0 × 10^−6^. The grid used in this test includes the channel flow field with a channel width of 2 mm, rib width of 2 mm, and channel height of 2 mm. As shown in Figure 5a, when the number of grid elements increased from approximately 1.4 million to 2.6 million, the error decreased to less than 0.1%, and the change in current density due to the increase in grid elements was determined to have converged. As illustrated in Figure 5b, three PTL thickness directional divisions were present, the element size was 0.182 mm, and subsequent simulations were performed on a grid at this level.

**Figure 6:**
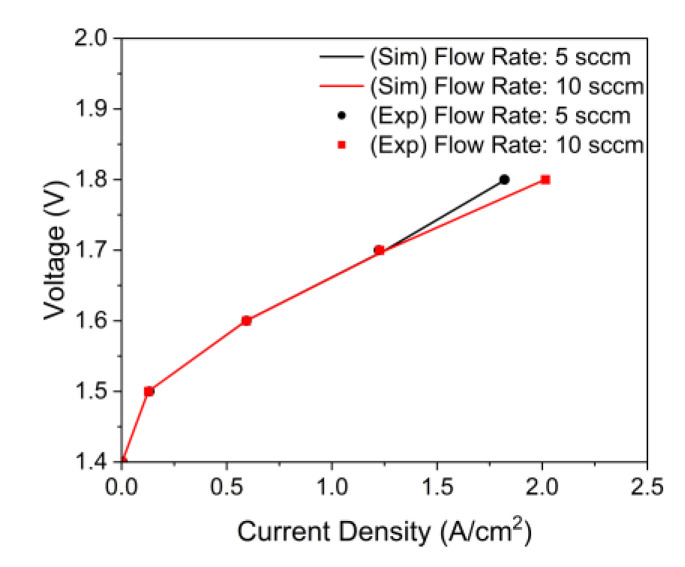
Comparison of polarization curves between simulation and experimental data by inlet flow rate (*W_Ch_*: 2 mm, *W_Rib_*: 2 mm, *H_Ch_*: 2 mm, *ε*: 0.75, *K*: 30.2 × 10^−12^ m^2^, *θ*: 30°, *T_in_*: 328.15 K, *T_cell_*: 328.15 K and $\vec{g}$: z+).

As shown in Figure 6, we compared the polarization curves based on the inlet flow rate of the simulation and experimental data. The inlet flow rate of 5 sccm had a stoichiometric ratio of 4.46 in an active area of 100 cm^2^ with a current density of 2 A/cm^2^, and the inlet flow rate of 10 sccm had a stoichiometric ratio of 8.92. When the inlet flow rate was reduced to 5 from 10, the current density decreased rapidly at a high operating voltage, and the polarization curve results of the simulation agreed well with the experimental data.

### 3.2. Effects of the Flow Field

**Figure 7:**
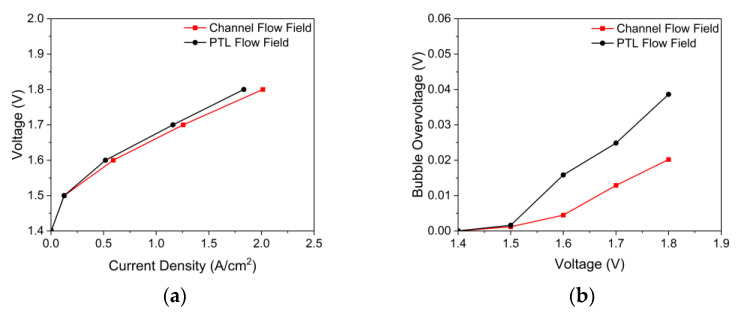
(**a**) Polarization curves and (**b**) bubble overvoltage curves of PEMWE according to the anode flow field curves (*ε*: 0.75, *K*: 30.2 × 10^−12^ m^2^, *θ*: 30°, ${\dot{Q}}_{in}$: 10 sccm, *T_in_*: 328.15 K, *T_cell_*: 328.15 K, and $\vec{g}$: z+).

The channel flow field had the following geometries *W_Ch_*: 2 mm, *W_Rib_*: 2 mm, *H_Ch_*: 2 mm, and the PTL flow field, *H_Ch_*: 0 mm. The effects of flow fields on the polarization are shown in Figure 7a. When the operating voltage was 1.5 V in the I–V curves, a slight difference existed in the current density according to the application of the flow field, but when the operating voltage was 1.8 V, the current density difference was approximately 10%. As shown in Figure 7b, this difference in performance was mainly due to the bubble overvoltage. The bubble overvoltage increased as the operating voltage increased, and at an operating voltage of 1.8 V, the bubble overvoltage was 0.02 V when the channel flow field was used, which was approximately 48% lower than when the PTL flow field was used. To better understand the increase in the performance with the flow fields, the pressure of the cell, water saturation on the active area, and current density distribution scene were obtained.

**Figure 8:**
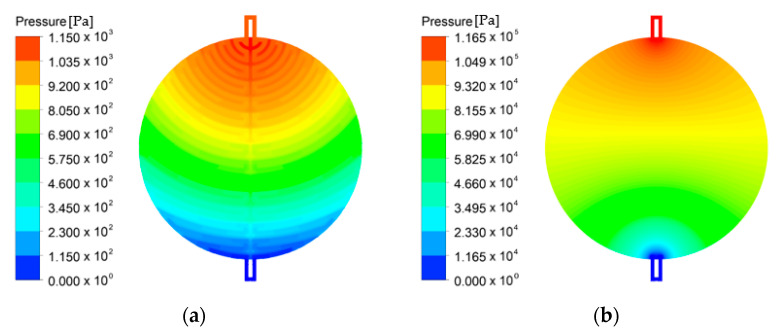
Pressure distribution inside the PEMWE cell using (**a**) the channel flow field and (**b**) the PTL flow field (*ε*: 0.75, *K*: 30.2 × 10^−12^ m^2^, *θ*: 30°, ${\dot{Q}}_{in}$: 10 sccm, *T_in_*: 328.15 K, *T_cell_*: 328.15 K, *V_op_*: 1.8 V and $\vec{g}$: z+).

The pressure distribution inside the cell with the channel flow field is illustrated in Figure 8a. The required pressure drop in the channel flow field was approximately 1 kPa. In addition, a pressure drop gradually appeared from the inlet to the outlet, but not symmetrically. The pressure drop in the cell gradually appeared under the influence of the channel shape. The channel shape of this flow field intentionally formed a small pressure drop from the inlet side and a large pressure drop from the outlet side. Owing to this channel shape, the water saturation near the outlet did not decrease and the current density was uniform. As illustrated in Figure 8b, the pressure drop was slightly greater than 100 kPa in the PTL flow field. In addition, the pressure decreased gradually and symmetrically from the inlet to the outlet. The required pressure drop increased when the flow inside the cell only flowed through the PTL because of low permeability. 

**Figure 9:**
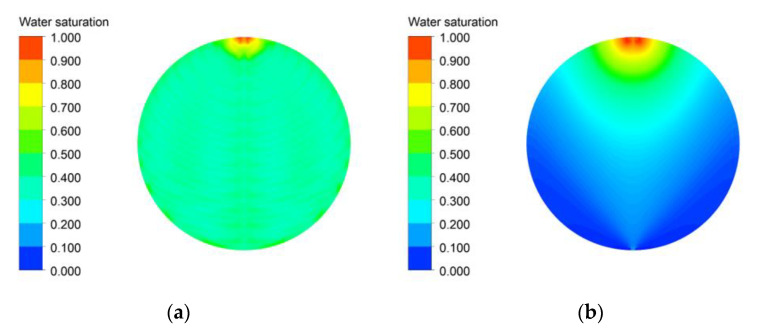
PEMWE’s water saturation distribution on the active area with (**a**) the channel flow field and (**b**) the PTL flow field (*ε*: 0.75, *K*: 30.2 × 10^−12^ m^2^, *θ*: 30°, ${\dot{Q}}_{in}$: 10 sccm, *T_in_*: 328.15 K, *T_cell_*: 328.15 K, *V_op_*: 1.8 V and $\vec{g}$: z+).

A clear difference in the water saturation distribution on the active area of channel and PTL flow fields is shown in Figure 9. For the channel flow field in Figure 9a, the water saturation did not decrease gradually from the inlet to the outlet, but generally remained uniform. Through the channel, the oxygen generated by the reaction moved to a nearby channel and was removed from the active area without getting accumulated in the PTL, and water was properly supplied from the channel to the active area. As shown in Figure 9b, The water saturation gradually decreased from the inlet to the outlet in the case of the PTL flow field, and the zone in which the water saturation was lower than 0.1 was found near the outlet. Oxygen generated by the reaction could not flow up to the channel without channel and was accumulated in the pores by flowing through the PTL. In addition, when the water saturation was lower than 0.1, the bubble overvoltage increased rapidly, which significantly affected the current density. 

**Figure 10:**
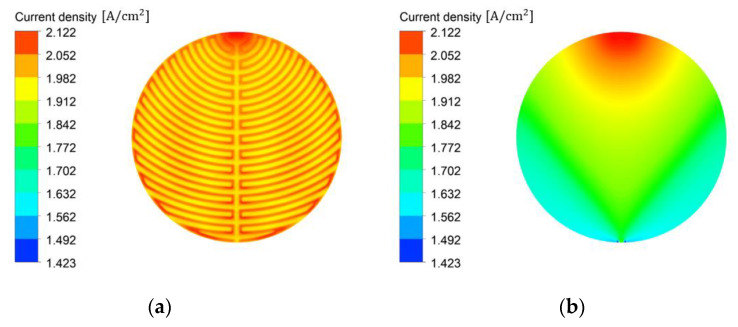
Current density distribution on the active area using (**a**) the channel flow field and (**b**) the PTL flow field of PEMWE (*ε*: 0.75, *K*: 30.2 × 10^−12^ m^2^, *θ*: 30°, ${\dot{Q}}_{in}$: 10 sccm, *T_in_*: 328.15 K, *T_cell_*: 328.15 K, *V_op_*: 1.8 V and $\vec{g}$: z+).

Figure 10a illustrates the effects of the channel flow field on the current density distribution on the active area of PEMWE. The current density distribution was high and uniform on the active area with the channel flow field. Therefore, if water saturation is high and uniform on the active area, a high and uniform current density can be obtained on the active area. The effects of the PTL flow field on the current density distribution is shown in Figure 10b. In the PTL flow field, the current density distribution near the outlet was low and more nonuniform than that in the channel flow field because the current density was low in the region with decreased water saturation. If the current density is not uniform on the active area and decreases from the inlet toward the outlet, the heat flux generated by the current density is also reduced, and such a deviation negatively affects the life and performance of the PEMWE. 

### 3.3. Effects of the Channel Geometry

**Figure 11:**
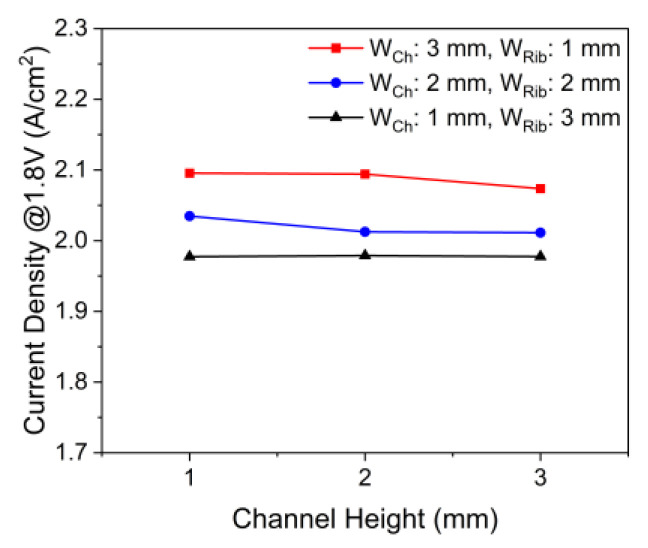
Effects of channel width-to-rib width ratio and channel height on PEMWE’s current density (*ε*: 0.75, *K*: 30.2 × 10^−12^ m^2^, *θ*: 30°, ${\dot{Q}}_{in}$: 10 sccm, *T_in_*: 328.15 K, *T_cell_*: 328.15 K, *V_op_*: 1.8 V and $\vec{g}$: z+).

To investigate whether the performance can be improved by changing the channel geometry, the effect of the channel geometry on the performance at a fixed operating voltage was investigated. Figure 11 shows the effects of the channel width-to-rib width ratio and channel height on the current density at an operating voltage of 1.8 V. At all channel heights, the current density was the highest when the channel width-to-rib width ratio was 3:1, and when the channel width-to-rib width ratio was changed from 1:3 to 3:1, the current density increased up to approximately 6%. As the channel width-to-rib width ratio increased, the contacting area of the channel and PTL contact increased and the rib width reduced the distance required for water and oxygen to flow between the channel and active area under the rib. When the channel height was changed from 3 to 1 mm, the current density hardly reduced by 2%. A lower channel height decreased the volume of the channel but increased the flow rates of water and oxygen in the channel. Nevertheless, when the channel height eventually reached 0 mm, the performance decreased. 

**Figure 12:**
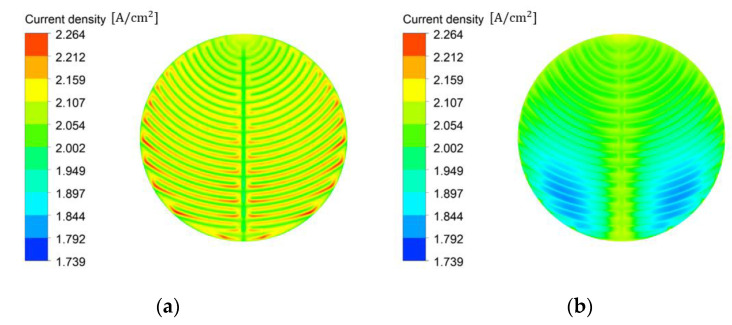
Current density distribution of PEMWE on the active area by channel width-to-rib width ratio (**a**) *W_Ch_*: 3 mm, *W_Rib_*: 1 mm, (**b**) *W_Ch_*: 1 mm, *W_Rib_*: 3 mm (*H_Ch_*: 1 mm, *ε*: 0.75, *K*: 30.2 × 10^−12^ m^2^, *θ*: 30°, ${\dot{Q}}_{in}$: 10 sccm, *T_in_*: 328.15 K, *T_cell_*: 328.15 K, *V_op_*: 1.8 V and $\vec{g}$: z+).

The current density distribution on the active area based on the channel width-to-rib width ratio at a channel height of 1 mm is illustrated in Figure 12a,b. When the channel width-to-rib width ratio was 3:1, the current density did not decrease because of the oxygen accumulated on the active area under the rib near the outlet. When the channel width-to-rib width ratio was 1:3, oxygen accumulated on the active area under the rib close to the outlet, which decreased the current density. Then, the surface-averaged data were obtained on the active area according to the channel width-to-rib width ratio. From the simulation results, the surface-averaged water saturation on the active area was 0.40, 0.45, and 0.49, when the channel width-to-rib width ratio was 1:3, 1, and 3:1, respectively. The surface-averaged bubble overvoltage on the active area with increasing water saturation was 0.021, 0.017, and 0.016 V, and the surface-averaged temperature on the active area was 329.4, 330.7, and 333.0 K for the channel width-to-rib width ratio of 1:3, 1, and 3:1, respectively. Owing to the increase in temperature, the areal resistance of the Nafion 117 membrane decreased slightly to 0.114, 0.112, and 0.109 ohm∙cm^2^, for the respective channel width-to-rib width ratio of 1:3, 1, and 3:1. Therefore, the current density improved and the bubble overpotential and areal resistance of the membrane decreased as the channel width-to-rib width ratio increased.

### 3.4. Effects of the PTL Properties

**Figure 13:**
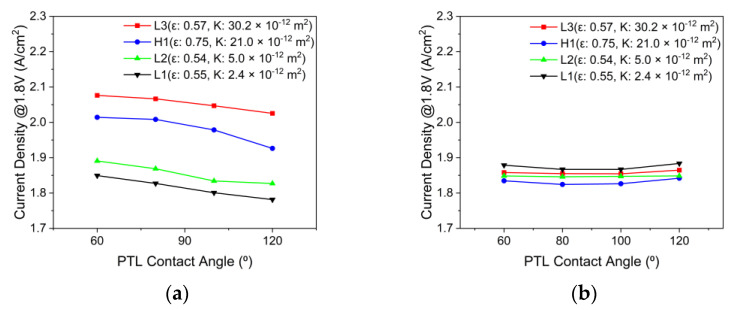
Effects of the PTL properties on PEMWE’s current density with (**a**) the channel flow field and (**b**) the PTL flow field (${\dot{Q}}_{in}$: 10 sccm, *T_in_*: 328.15 K, *T_cell_*: 328.15 K, *V_op_*: 1.8 V and $\vec{g}$: z+).

The effects of the PTL properties on PEMWE’s current density with the channel flow field are depicted in Figure 13a. The higher the permeability of the PTL at all contact angles, the better the performance, particularly when the PTL type changed from L1 to L3 at a contact angle of 60°, and the current density increased by approximately 12%. The higher the permeability, the lower the resistance of the water and oxygen flow inside the PTL. The performance improved when the contact angle changed from hydrophobic to hydrophilic, and in the case of PTL type L3, the current density increased by approximately 3% when the contact angle changed from 120° to 60°. Hydrophilic PTL enhances the attraction of water from the channel to the PTL at their interface, whereas hydrophobic PTL decreases the release of oxygen from the PTL to the channel at the interface between the PTL and channel. Figure 13b shows effects of the PTL properties on PEMWE’s current density with the PTL flow field. Unlike the case of the channel flow field, the performance did not improve as the PTL permeability increased. In order to understand the reason why the PTL type of L1, which has the lowest permeability, has increased performance, the capillary pressure for each PTL type was calculated.

**Figure 14:**
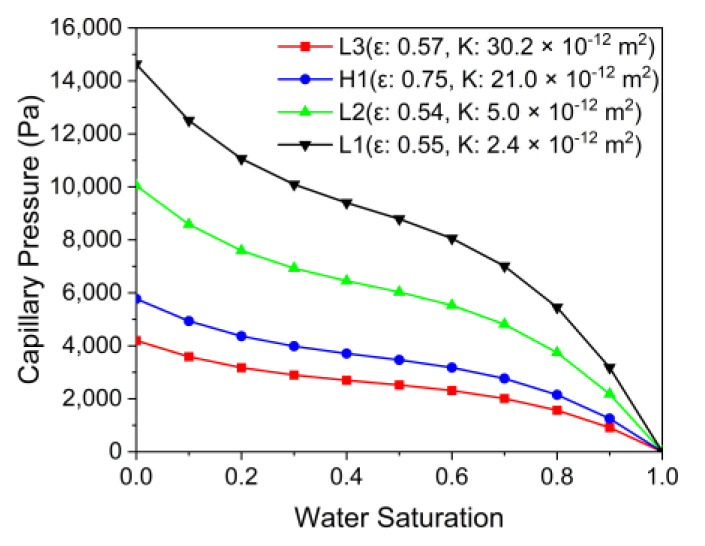
Capillary pressure saturation curves calculated as the Leverett J-function from 0 to 1 at every water saturation of 0.1 by the PTL types.

Figure 14 shows the capillary pressure saturation curves calculated as the Leverett J-function from 0 to 1 at every water saturation of 0.1 by PTL type. PTL type L1 showed a capillary pressure that was nearly three times that of L3. The lower the permeability, the higher the capillary pressure at the same saturation was calculated. Lower permeability increases resistance to the flow of water and oxygen, but capillary pressure increases at the same saturation, which is thought to have a positive effect on performance. Therefore, with the PTL flow field, finding the optimum point of the flow resistance and capillary pressure created by the PTL is important in order to improve the performance.

### 3.5. Effects of the Gravity

**Figure 15:**
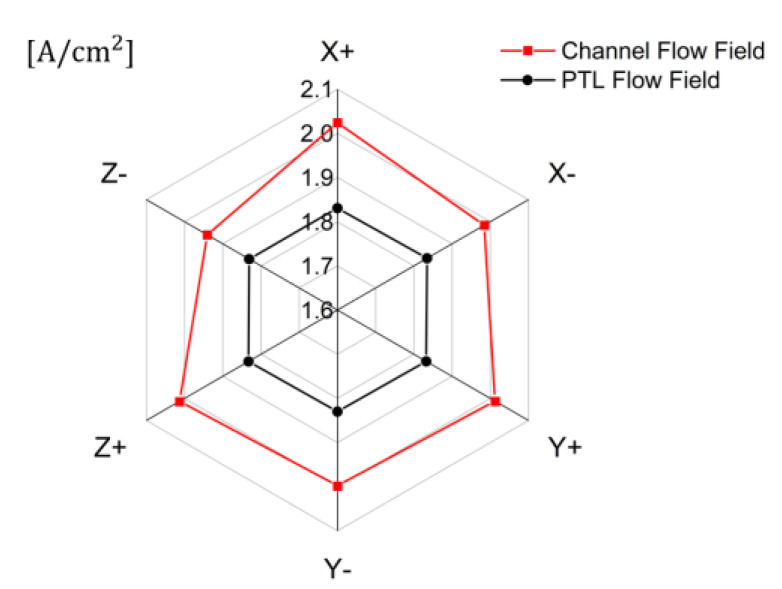
Effects of the gravity on current density with channel and PTL flow fields in PEMWE (*ε*: 0.75, *K*: 30.2 × 10^−12^ m^2^, *θ*: 30°, ${\dot{Q}}_{in}$: 10 sccm, *T_in_*: 328.15 K, *T_cell_*: 328.15 K, *V_op_*: 1.8 V).

The effects of gravity on the current density with channel and PTL flow fields at an operating voltage of 1.8V are shown in Figure 15. The direction of gravity only affects the performance with the channel flow field. The current density was the highest in the gravity directions of X+ and Z+ and was the lowest in the direction of gravity of Z-. In particular, the current density in the gravity direction X+ was approximately 4% higher than that in Z-. The direction of gravity on the cell changes the direction of the buoyancy received by oxygen inside the cell and affects the water saturation distribution inside the PTL and PEMWE performance. The direction of gravity X+ allows water inside the PTL to flow from the inlet to the outlet, Z+ allows water from the PTL to flow from the channel to the active area, improving performance, and the direction of gravity Z- reduces performance by allowing oxygen to flow from the channel to the active area. The pressure drop of the cell was more than 100 kPa with the PTL flow field, and the effect of gravity on the distribution of water saturation inside the PTL was less dominant than the pressure drop. Therefore, with the channel flow field, it should be operated considering the direction of gravity, and operating the cell in the direction of gravity of X+ or Z+ improves the performance when the pressure drop does not dominate the water saturation distribution.

### 3.6. Effects of the Flow Rate

**Figure 16:**
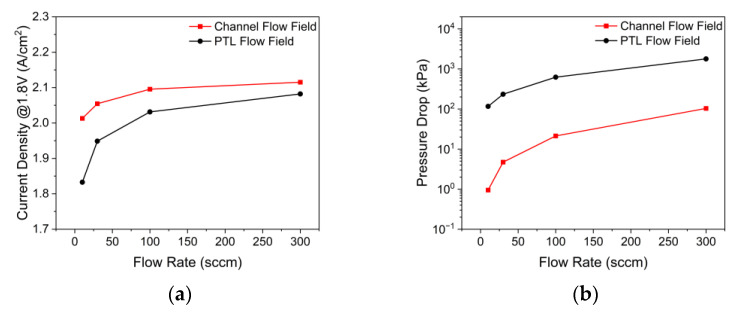
Effects of flow rate on the (a) current density and (b) pressure drop according to the anode flow field of PEMWE (*ε*: 0.75, *K*: 30.2 × 10^−12^ m^2^, *θ*: 30°, ${\dot{Q}}_{in}$: 10 sccm, *T_in_*: 328.15 K, *T_cell_*: 328.15 K, *V_op_*: 1.8 V).

The effects of the flow rate on the current density according to the anode flow field of PEMWE are shown in Figure 16a. When the inlet flow rate increased from 10 sccm to 300 sccm, the current density increased by 5% from 2.013 A/cm^2^ to 2.115 A/cm^2^ with the channel flow field, and when the inlet flow rate increased from 10 sccm to 300 sccm with the PTL flow field, the current density increased by 14% from 1.832 A/cm^2^ to 2.066 A/cm^2^. In addition, the difference in the current density between the two flow fields decreased from 10% to 2% when the inlet flow rate increased from 10 sccm to 300 sccm. As the inlet flow rate increased, the stoichiometric ratio increased and the water saturation on the active area increased, thereby reducing the bubble overvoltage. The current density at a constant operating voltage converged when the bubble overvoltage disappeared as the inlet flow rate increased. Next, the effects of the flow rate on the pressure drop according to the anode flow field of PEMWE are illustrated in Figure 16b. For the inlet flow rate of 300 sccm, the pressure drop was 103.67 kPa with the channel flow field, and 1784.34 kPa with the PTL flow field. The rate of increase in the pressure drop with the increasing inlet flow rate was greater in the case of the channel flow field, but the absolute pressure drop of the PTL flow field in this inlet flow rate range was always greater. Therefore, when the inlet flow rate increases, the performance difference according to the flow field can be reduced; however, a large pressure drop in the PTL flow field should be considered.

## 4. Conclusions

In this study, we used a three-dimensional two-phase model to analyze the electrochemical performance of the PEMWE. First, the possibility of a PEMWE with the PTL flow field was analyzed and compared to the channel flow field. Next, the effects of the channel geometry, PTL properties, gravity, and flow rate were investigated. The important findings are as follows:

1. The main factor in the performance differences is the bubble overvoltage caused by the lower water saturation on the active area. A lower stoichiometric ratio increased the bubble overvoltage, and the polarization curve results of the simulation and experiment showed high consistency.
2. At a low stoichiometric ratio, a high and uniform current density was obtained using the channel flow field. Moreover, the performance can be improved by reducing the distance between the channel and area under the rib where the water and oxygen must flow, reducing the resistance of the flow in the PTL. The direction of gravity helped improve performance when setting the direction to help water flow from the channel to the active area or from the inlet to the outlet.
3. At a high stoichiometric ratio, the performances of channel and PTL flow fields become similar because of the gradually disappearing bubble overvoltage. However, a significantly large pressure drop must be considered in the PTL flow field.

Finally, the limitation of this study is that transient phenomena in the formation and change of oxygen bubbles were not considered. For a better understanding of the causes of bubble overvoltage, transient simulations of oxygen bubbles can be studied later. In addition, future studies can also be conducted to see how the performance of PEMWE is affected by lower channel heights, larger channel width-to-rib width ratios, and both.

## Figures and Tables

**Table 1:** The geometric parameters of the flow field used in the PEMWE simulation with the two-phase flow model.

Parameter	Symbol	Value
Channel width	$W_{Ch}$	1 mm, 2 mm, 3 mm
Rib width	$W_{Rib}$	3 mm, 2 mm, 1 mm
Channel height	$H_{Ch}$	1 mm, 2 mm, 3 mm
PTL thickness	$t_{PTL}$	0.25 mm

**Table 2:** Parameters used in the electrochemical model of the PEMWE.

Parameter	Symbol	Value
Reversible voltage in standard state	$E^{0}$	1.229 V
Amount of change in entropy	Δs	163.23 J/K
Faraday’s constant	F	96,485 C/mol
Temperature in standard state	$T_{0}$	298.15 K
Universal gas constant	R	8.314 J/K∙mol
Tafel coefficient	αn	1.328
Exchange current density	$i_{0}$	1.6 × 10^−3^ A/cm^2^
Membrane thickness	$t_{mem}$	0.175 mm
Water content	λ	22

**Table 3:** Parameters used in the conservation equations of the PEMWE model.

Parameter	Symbol	Value
Water density	$\rho_{l}$	985.64 kg/m^3^
Oxygen density	$\rho_{g}$	1.191 kg/m^3^
Water viscosity	$\mu_{l}$	0.000504 kg/m∙s
Oxygen viscosity	$\mu_{g}$	0.000022 kg/m∙s
Surface tension coefficient	σ	0.063 N/m
Gravity direction	$\vec{g}$	X+, X-, Y+, Y-, Z+, Z-

**Table 4:** PTL properties in the PEMWE simulation.

Parameter	Symbol	Value			
Type		L1	L2	L3	H1
Porosity	ε	0.55	0.54	0.57	0.75
Permeability	K	2.4 × 10^−12^ m^2^	5.0 × 10^−12^ m^2^	21.0 × 10^−12^ m^2^	30.2 × 10^−12^ m^2^
Contact angle	θ	30°, 60°, 80°, 100°, 120°			

**Table 5:** Parameters used in the boundary conditions of the PEMWE model.

Parameter	Symbol	Value
Inlet water flow rate	${\dot{Q}}_{in}$	5 sccm, 10 sccm, 30 sccm, 100 sccm, 300 sccm
Inlet water temperature	$T_{in}$	328.15 K
Outlet pressure	$P_{out}$	0 Pa
Cell temperature	$T_{cell}$	328.15 K
Water molecular weight	$M_{l}$	18 kg/kmol
Oxygen molecular weight	$M_{g}$	32 kg/kmol
Operating voltage	$V_{Op}$	1.4 V, 1.5 V, 1.6 V, 1.7 V, 1.8 V
Thermal neutral voltage	$V_{Th,neu}$	1.48 V

## Data Availability

Not applicable.
